# Enteroendocrine peptides, growth, and the microbiome during the porcine weaning transition

**DOI:** 10.1186/s42523-022-00206-8

**Published:** 2022-11-18

**Authors:** T. G. Ramsay, A. M. Arfken, K. L. Summers

**Affiliations:** grid.507312.2 0000 0004 0617 0991 U.S. Department of Agriculture, Agricultural Research Service, Beltsville Agricultural Research Center, Beltsville, MD 20705 USA

**Keywords:** Piglet, Weaning, Microbiome, Bacteriome, Enteroendocrine, GLP-1, GLP-2, GIP, IGF-1

## Abstract

**Background:**

Growth rate in pigs can be affected by numerous factors that also affect feeding behavior and the microbiome. Recent studies report some communication between the microbiome and the enteroendocrine system. The present study examined if changes in the piglet microbiome between birth and during the weaning transition can be correlated either positively or negatively with growth rate and plasma concentrations of enteroendocrine peptides.

**Results:**

During the post-weaning transition, a 49% reduction in average daily gain was observed at day 24 (*P* < 0.05) relative to day 21. Pigs recovered by day 28 with body weight and average daily gain increases of 17% and 175%, respectively relative to day 24 and the highest rate of gain was measured at day 35 (462 g/day). The time interval between day 21–24 had the highest number of correlations (n = 25) between the relative abundance differences in taxa over time and corresponding percent weight gain. Amplicon sequence variants with the greatest correlation with percent weight gain between day 21–24 belonged to families *Prevotellaceae* NK3B31 (ρ = 0.65, *P* < 0.001), *Veillonellaceae* (ρ = 0.63, *P* < 0.001) and *Rikenellaceae* RC9 (ρ = 0.62, *P* < 0.001). Seven taxa were positively correlated with percent weight gain between day 24–28. Eight taxa were positively correlated with percent weight gain between day 28–35, of which four were Clostridia. Only *Lactobacillus reuteri* was positively correlated across both day 24–28 and day 28–35 analyses. Insulin-like growth factor 1 (IGF-1; R2 = 0.61, *P* < 0.001), glucose-dependent insulinotropic polypeptide (GIP; R2 = 0.20, *P* < 0.001), glucagon-like peptide 1 (GLP-1; R2 = 0.51, *P* < 0.001), and glucagon-like peptide 2 (GLP-2; R2 = 0.21, *P* < 0.001) were significantly associated with the piglet fecal community NMDS, while serotonin showed no significant association (R2 = 0.03, *P* = *0.15*). Higher concentrations of GLP-1 and GLP-2 characterized day 1 fecal communities, while GIP levels had the strongest relationship primarily with samples ordinated with the day 21 cluster.

**Conclusions:**

Demonstration of an association of certain taxa with individual gut peptides at specific ages suggests the potential for the microbiome to elicit changes in the gut enteroendocrine system during early postnatal development in the pig.

**Supplementary Information:**

The online version contains supplementary material available at 10.1186/s42523-022-00206-8.

## Introduction

The gut of the healthy neonatal pig experiences an initial microbial colonization at birth with the adaptation to the external environment in combination with the introduction of a milk-based diet. At weaning, the subsequent transition to solid food imparts another transition for the gut microbiome. Several studies in swine have demonstrated that the physical and metabolic/biochemical interactions of the microbiome with the early postnatal diet result in changes in the relative contribution of various bacterial families to the overall microbial ecology of the host environment [1–5].

How the microbiome interacts with the physiology of the neonatal animal to elicit changes in growth rate is of current interest. Several studies have demonstrated that changes in the microbiome elicit changes in short chain fatty acid production and affect intestinal growth and barrier function [4, 6, 7]. Additionally, interactions of the microbial community with the gut wall result in changes in cytokine and growth factor expression by intestinal cells [8, 9]. These cytokines and growth factors may potentially impact feeding behavior, peripheral metabolism and consequently growth rate.

Growth rate can be affected by environment, diet, behavior and stress (psychological and physiological). Mediators for many of these factors that can impact growth rate can also affect feeding behavior, directly or indirectly, and thus potentially affect the microbiome [2, 10, 11]. Studies with post-weaning pigs have demonstrated a relationship between the microbiome and feed efficiency, therefore growth rate [12]. The interaction of the microbiome with growth rate during the weaning transition has not been thoroughly evaluated. The interaction of the microbiome with feed efficiency and growth rate is most likely through short chain fatty acids, biogenic amines, neuroendocrine secretions, and the enteroendocrine system [13, 14]. Recent work has demonstrated interactions exist between the microbiome and the enteroendocrine system [15, 16]. For example, the bacterial metabolite and quorum sensing molecule indole can stimulate glucagon-like peptide 1 (GLP-1) secretion into the vasculature [17]. Germ free mice have reduced enterochromaffin cell (EC) seratonin levels, while colonization of the gut with donor microbiota restores seratonin levels in EC cells [18]. Butyrate, the bacterial metabolites and short chain fatty acid has been reported to stimulate glucose-dependent insulinotropic polypeptide (GIP) secretion following oral administration [19].

The enteroendocrine system produces a variety of gut peptides. Several of these have been reported to impact feed intake, peripheral tissue metabolism and tissue accretion. Many of these actions are through receptors present on vagal afferents; however, some responses are direct effects of these molecules in the periphery. Gut serotonin enters the bloodstream and promotes insulin secretion in the pancreas and stimulates lipogenesis in adipose tissue and liver, while inhibiting lipolysis [20]. GIP has been demonstrated to stimulate lipid uptake and storage in adipocytes while inhibiting lipolysis and is widely considered an adipogenic hormone [21]. GLP-1 is an incretin hormone that stimulates insulin secretion and inhibits glucagon release from the pancreas [22]. Recently, GLP-1 was reported to alter skeletal muscle remodeling by altering glucose uptake, mitochondrial biogenesis and type 1 fiber formation [23]. GIP, GLP-1 and glucagon-like peptide-2 (GLP-2) inhibit bone resorption and thus may impact bone remodeling [24]. These various actions of the gut peptides suggest they may have important roles in growth.

The present study examined if changes in the piglet microbiome between birth and the adaptation to solid feed during the weaning transition can be correlated either positively or negatively with growth rate and plasma concentrations of the enteroendocrine peptides GIP, GLP-1, GLP-2 and serotonin.

## Materials and methods

### Animals

Large White × Landrace piglets from 8 litters (L.1-8) were assessed from birth through day 35 of age and were weaned at day 21. Piglets were not provided milk replacer/supplement or creep feed at any point throughout the experiment. The diets were formulated to meet the National Research Council estimate of nutrient requirements. From days 21–28, piglets received Nursery Diet 1 exclusively, followed by Nursery Diet 2 exclusively from days 29–35 (Additional file 1). Piglets were given free access to feed and water, evaluated daily for health, and weights were recorded for each sample time point. Animal health was evaluated by veterinary staff. A healthy state was defined by weight gain trends, lack of respiratory issues, absence of scabbing or wounds, alertness. All piglets used in this study were observed to be healthy. No antibiotics, antifungals, or supplemental additives were administered to the piglets at any time during the experiment. Care and treatment of all pigs were approved by the USDA-ARS Institutional Animal Care and Use Committee of the Beltsville Agricultural Research Center.

### Fecal sample collection

Fresh fecal samples were collected into sterile cryovial tubes from the rectum of individual piglets (n = 48) on days 1, 21 (weaning), 24, 28 and 35. Samples were initially flash frozen in liquid nitrogen and stored at − 80 °C until further processing. A total of 243 fecal samples were collected.

### Blood collection

Blood (0.5–1.5 mL) was collected from the cranial vena cava, and plasma was harvested following centrifugation of EDTA-treated syringes at days 1, 21, and 35 of age with S-Monovette K3E (Sarstedt, Nümbrecht, GE). Plasma samples were stored at − 20 °C until analyzed.

### ELISA

Plasma insulin-like growth factor 1 (IGF-I, ALPCO, Salem, NH, 03079), serotonin (5HT, Enzo Life Sciences Inc., Farmingdale, NY 11735), gastric inhibitory peptide (GIP, Millipore, Billerica, MA 01821), glucagon-like peptide 1 (GLP-1, Millipore, Billerica, MA 01821) and glucagon-like peptide 2 (GLP-2, AssayPro LLC, St. Charles, MO, 63301) were analyzed with commercial ELISA kits according to the manufacturer’s instructions. A standard curve was prepared according to manufacturer’s recommendations using a PL4 log-transformed sigmoidal algorithm (SigmaPlot 14.0, Systat Software Inc., Chicago, IL); unknowns were extrapolated from that standard curve.

### DNA extraction and sequencing

DNA was isolated from 0.25 g feces using the MagAttract Power Microbiome Kit (Qiagen, Hilden, Germany) by the Microbial Systems Molecular Biology Laboratory at the University of Michigan. Cells were lysed to isolate DNA using mechanical bead beating for 20 total minutes with 20 frequency/second and extracted using magnetic bead technology according to the Qiagen protocol. The V4 region of the 16S rRNA-encoding gene was amplified from extracted DNA using the barcoded dual-index primers developed previously [25]. 16S regions were sequenced with the Illumina MiSeq Sequencing platform generating 2 × 250 bp paired-end reads. All sequences are publicly available in the NCBI’s sequence read archive (SRA) under BioProjects PRJNA613280 and PRJNA673841.

### Sequence processing

Quality filtering, pairing, denoising, amplicon sequence variants (ASVs) determination, and chimera removal was conducted with the DADA2 v. 2020.8.0 [26] plugin in QIIME2 v. 2020.4 [27]. For quality trimming, reverse reads were truncated to 200 bp. Taxonomic classification of the ASVs was performed using the Qiime2 pretrained 16S 515F/806R [28–30] from the Silva 138 database [31]. Samples with < 5000 sequences (n = 7) and ASVs identified as archaea, chloroplast, mitochondria, or unassigned were removed. A total of 10,039,464 sequences with an average of 41,315 ± 1919 reads per sample (n = 243) were selected for downstream analysis.

### Statistical analysis

Assumptions of samples’ independence, normal distribution and equal variances have been verified for all variables. Equality of variances was tested using Levene’s test in R 4.1.3 (https://r-project.org/). When the data were not normally distributed and the assumption of equal variance has been rejected (serotonin and GLP-1) two-way non-parametric ANOVA (Scheirer–Ray–Hare test) was performed with Dunn’s test as post-hoc analysis (Kruskal–Wallis multiple comparison with *p* values adjusted with Benjamin-Hochberg method) in R. Data characterized by normal distribution and equal variances were analyzed by two-way ANOVA using the GLM procedure in SAS (SAS, Inc., Cary, NC). Least significant differences were used to determine which means differed significantly. The model included the main effects of age, sex, pig and sow. Sex and pig had no effect (*P* > 0.05) for any of the measured parameters, so the only two-way interaction tested was age (or age range for ADG) x sow.

Non-metric multidimensional scaling (NMDS) of piglet fecal communities was performed using the R-packages vegan (https://CRAN.R-project.org/package=vegan) and phyloseq [32] with the metaMDS function (k = 2, autotransform = False, trymax = 1000) on cumulative sum scaling transformed (CSS) ASVs and Bray–Curtis dissimilarity distances. Permutational analysis of variance (PERMANOVA) was used to determine the main effect of growth time points on the piglet fecal microbial communities using the adonis function (permutations = 1000) in vegan. The strata option (strata = piglet) was used to account for repeated sampling of individual piglets. ASVs present < 1% of samples were removed prior to analysis to reduce noise and the occurrences of rare ASVs. Concentrations of serotonin, GIP, GLP-1, and IGF-1 concentrations were log transformed; a pseudocount of 1 was added to GLP-2 log transformations to prevent negative values. The envfit function (perm = 999) in vegan was then used to fit peptide vectors to the piglet fecal community ordination plot using regression and was visualized using ggordiplots (http://github.com/jfq3/ggordiplots) and ggplot2 packages [33]. Interaction network analyses among centered-log-ratio transformed (CLR) ASVs and log transformed peptide concentrations at each time point were conducted with the Julia based FlashWeave program (sensitive = true, heterogenous = false) [34] which uses a local-to-global learning approach to test for conditional independence and predict direct relationships among microbial taxa and metadata variables. Prior to transformations and network analysis, ASVs in < 30% of samples were filtered from samples to remove rare ASVs and avoid overabundance of zeroes in log ratio transformations, which can lead to spurious results. Network plots were visualized using the Cytoscape v. 3.8.0 [35]. Spearman rank correlations between relative abundance changes in ASVs and percent weight gain in piglets over consecutive time points were performed on total sum scale normalized (TSS) ASVs using the function rcorr (type = “spearman”) in the R-package corrplot (https://github.com/taiyun/corrplot). To reduce correlation bias of zero-inflated data, ASVs present < 50% of the samples at each time point comparison were removed from analysis. Corresponding *P* values were calculated using the function cor.test and corrected for the false discovery rate (FDR). Correlation plots were visualized using the corrplot function. To visualize relationships between taxa, a neighbor-joining phylogenetic tree [36] based on the multiple sequence comparison by the log-expectation [37] aligned V4 region of 16S rRNA genes were constructed in MEGAX [38]. All analyses in this study used a significance threshold of *P* < 0.05.

## Results

### Animal weight gain with age

**Fig. 1:**
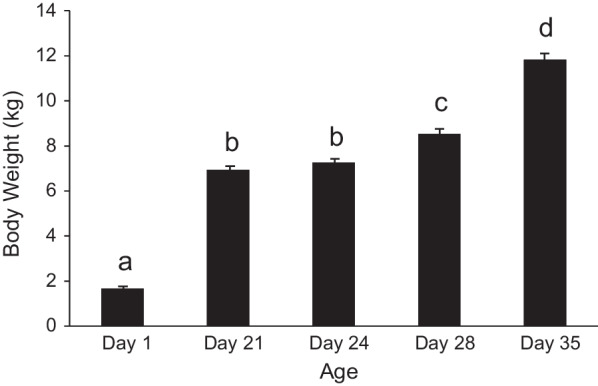
Body weights of pigs from birth to day 35 of age. Legend: Bars represent means and error bars represent standard errors. Means not sharing a common superscript letter are significantly different*.* Significant age effect (*P* < 0.05), sow effect (*P* < 0.0001) and age × sow effect (*P* < 0.0001), n = 48

Pigs used in this study were born at an average birth weight of 1.66 ± 0.04 kg (Fig. 1). Weaning at day 21 of age was accompanied by a mean body weight of 6.94 ± 0.16 kg and an average daily gain (ADG) of 248 ± 8 g/d between day 1 and day 21 of age (Table 1). Weaning resulted in a 49% reduction in average daily gain at day 24 (*P* < 0.05) relative to day 21 and no change in body weight (*P* > 0.05). Body weight and average daily gain increased 17% and 175%, respectively, at day 28 relative to day 24 (*P* < 0.05). The heaviest body weight was observed at day 35 and accompanied by a further increase of 32% in average daily gain between day 28 and day 35 relative to day 24 to day 28 (*P* < 0.05).

**Table 1:** Average daily gain

Age range	ADG (grams/d)^1^	
(Days)	Mean	SE^2^
1–21	248^b^	8
21–24	127^a^	20
24–28	350^c^	12
21–28	254^b^	8
28–35	462^d^	15

^a,b,c,d^Means not sharing a common superscript letter are different for age (n = 48, *P* < 0.0001)

^1^Significant age effect (*P* < 0.0001), sow effect (*P* < 0.0001) and age × sow effect (*P* < 0.0001)

^2^SE = Standard error

### Plasma peptide concentrations

**Fig. 2:**
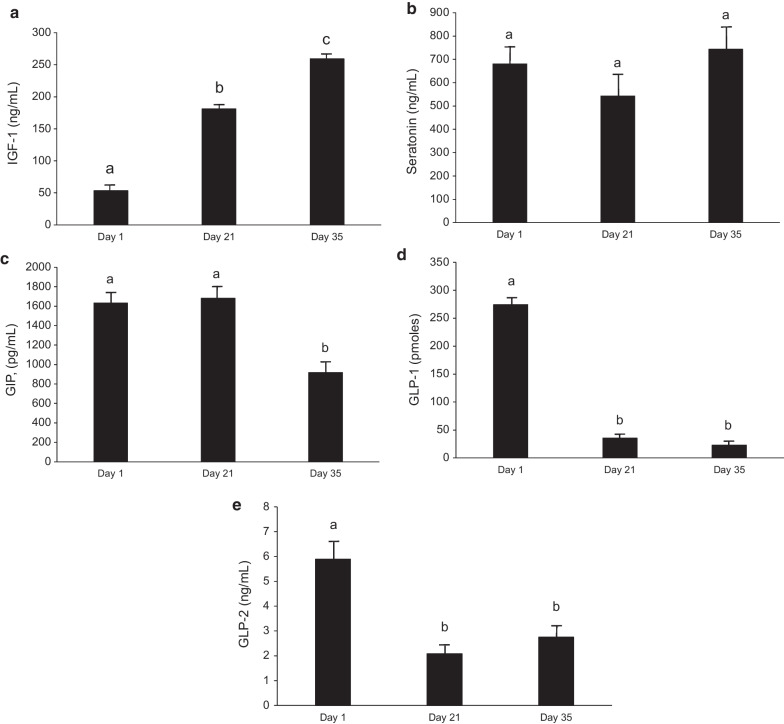
Plasma peptide concentrations in pigs at day 1, day 21 and day 35 of age. **a** Plasma Insulin-like growth factor 1 (IGF-1). Significant age effect (*P* < 0.0001); significant sow effect (*P* < 0.0001). **b** Plasma Seratonin. Significant sow effect (*P* < 0.0001) and age × sow effect (*P* < 0.0001). **c** Plasma Gastric inhibitory peptide (GIP). Significant age effect (*P* < 0.0001), sow effect (*P* < 0.0001) and age × sow effect (*P* < 0.0001). **d** Plasma Glucagon-like peptide 1 (GLP-1). Significant age effect (*P* < 0.05), sow effect (*P* < 0.01) and age × sow effect (*P* < 0.01). **e** Plasma Glucagon-like peptide 2 (GLP-2). Significant age effect (*P* < 0.0001) and age × sow effect (*P* < 0.001). Legend: Bars represent means and error bars represent standard errors. Means not sharing a common superscript letter are significantly different for age (n = 48; *P* < *0.05*)

Analysis of plasma peptides demonstrated an age-related increase in IGF-1 from day 1 to day 35 (Fig. 2a, *P* < 0.001). In contrast, no change in plasma seratonin was detected with age (Fig. 2b, *P* > 0.05). Plasma GIP did not differ at day 1 and day 21 of age (*P* > 0.05) but declined by 46% at day 35 (Fig. 2c, *P* < 0.01). Plasma GLP-1 concentration was 274 ng/mL at d1 in these pigs and dropped by 87–35 ng/mL at day 21, but with no further change at day 35 of age (Fig. 2d, *P* < 0.05). The plasma concentration of GLP-2 also declined between day 1 and day 21 (Fig. 2e, *P* < 0.001), but with no further change at day 35 (*P* > 0.05). Only plasma IGF-1 demonstrated a correlation with body weight in these pigs between day 1 and day 21 (R2 = 0.422, *P* < 0.0001) or day 1 and day 35 of age (R2 = 0.633; *P* < 0.001) of the plasma peptides quantified. Average daily gain was also correlated with plasma IGF-1 between day 1 and day 21 (R2 = 0.247, *P* < 0.001).

### Correlation between ASVs and weight gain over the weaning transition

ASV analysis did not detect any significant changes in the microbiome between day 1 and day 21 of age. However, Spearman Rank correlations between weight gain and the microbiome revealed that *Clostridium scindens* was unique (among those bacteria detected by 16S rRNA analysis) in being associated with growth in the preweaning pig (r = 0.604, *P* = 0.00004, FDR = 0.00119).

**Fig. 3:**
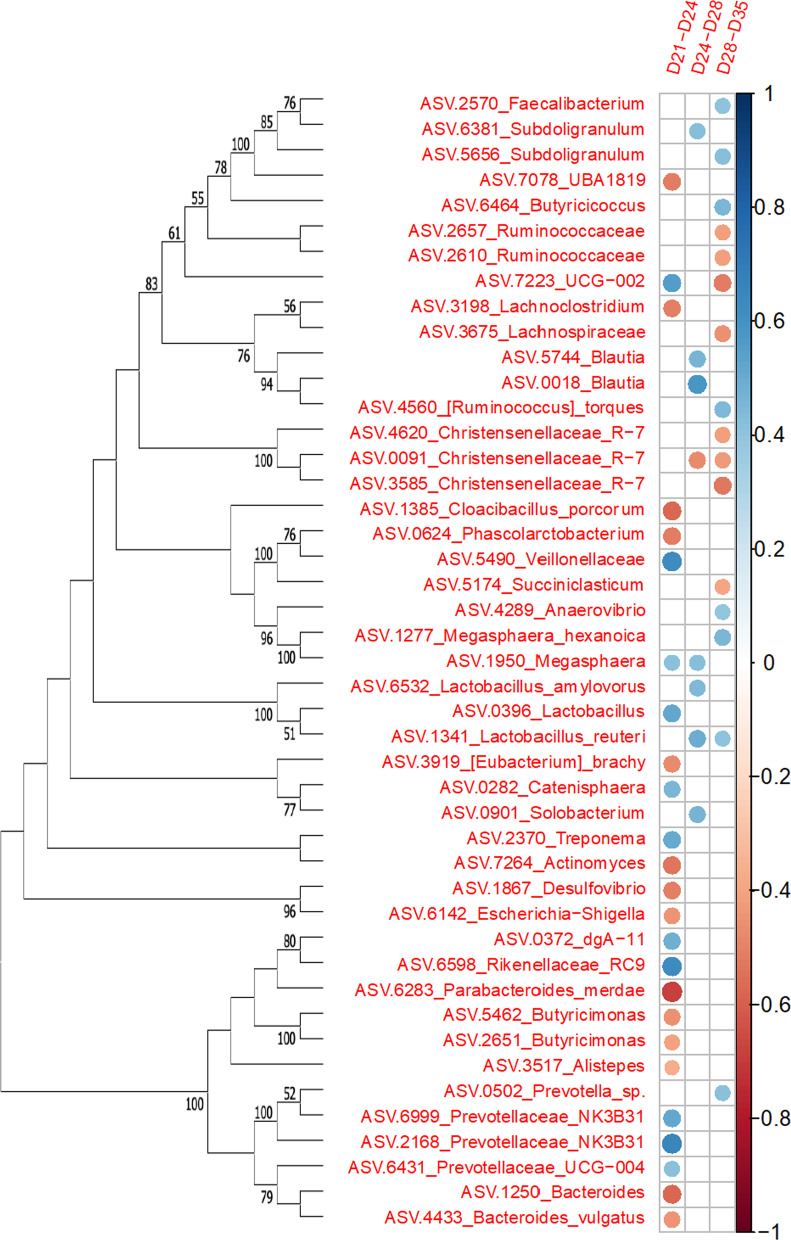
Correlations between weight gain and ASVs. Legend: Spearman rank correlation plot showing significant correlations (*P* < 0.05, FDR corrected) between change in percent relative abundance and percent relative weight gain between time points (D21-D24, D24-D28 and D28-D35). Red circles indicate negative and blue circles indicate positive correlations. The size of the circle indicates the strength of the correlation. Neighbor-joining tree with bootstrap values (bootstrap = 100, values < 50 not shown) based on MUSCLE alignment of 16S rRNA v4 gene region depict phylogenetic relationship between ASVs

The time interval from day 21 to day 24 had the highest number of significant correlations (n = 25) between the relative abundance differences in taxa over time and corresponding percent weight gain compared to time periods from day 24 to day 28 (n = 8) and from day 28 to day 35 (n = 16; Fig. 3). Between day 21 and day 24, increased relative abundances of ASVs belonging to families *Prevotellaceae* NK3B31 ($\rho$ = 0.65, *P* < 0.001), *Veillonellaceae* ($\rho$ = 0.63, *P* < 0.001) and *Rikenellaceae* RC9 ($\rho$ = 0.62, *P* < 0.001) were significantly correlated with an increase in percent weight gain. In contrast, increased relative abundances of ASVs *Parabacteroides merdae* ($\rho$ = − 0.58, *P* < 0.001), *Bacteroides* ($\rho$ = − 0.58, *P* < 0.001) and *Cloacibacillus porcorum* ($\rho$ = − 0.57, *P* < 0.001) were significantly correlated with a decrease in percent weight gain. *Bacteroidia*, *Clostridia* and *Negativicutes* accounted for 19 of the 25 identified genera or species that affected ADG between day 21 and day 24. Throughout the weaning transition, increased relative abundances of ASVs belonging to the *Lactobacillus* genera all showed significant and positive correlations with weight gain, while increased relative abundances of ASVs belonging to the *Christenellaceae* R7 group were significantly and negatively correlated with percent weight gain. ASV UCG-002 showed a significant positive correlation with percent weight gain between day 21 and day 24 ($\rho$ = 0.55, *P* < 0.001) but a significant negative correlation with percent weight gain between day 28 and day 35 ($\rho$ = − 0.52, *P* = 0.01). Seven bacterial genera or species were positively correlated with percent weight gain between day 24 and day 28, with only one genus correlating with percent weight gain for both day 21 to day 24 and day 24 to day 28: *Megaspaera*. Analysis of the correlations from day 28 to day 35 indicated the increased relative abundances of eight taxa that were positively correlated with percent weight gain, of which four were *Clostridia*. Only *Lactobacillus reuteri* was positively correlated across both day 24 to day 28 and day 28 day 35 analyses. Among the eight taxa that were negatively correlated with percent weight gain in the day 28 to day 35 analysis, all but *Succiniaclasticum* were of the *Clostridia* class.

### Piglet fecal community structure and peptides associated with age

**Fig. 4:**
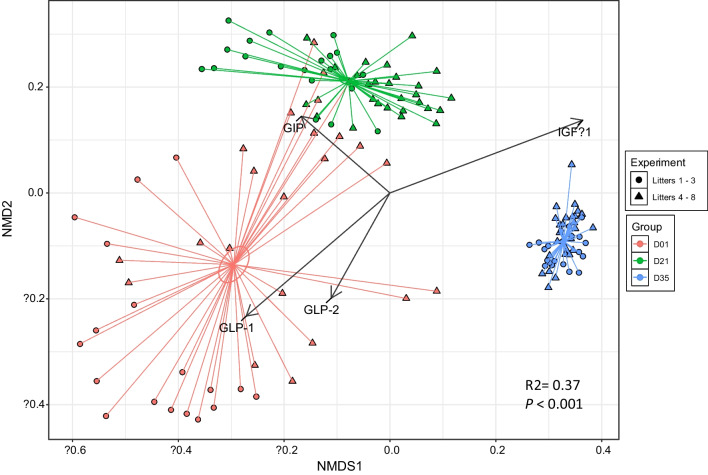
Non-metric multidimensional scaling (NMDS) plot of the piglet fecal community structure at different time points based on Bray–Curtis dissimilarity matrix. Legend: The effect of age on community was tested using permutational analysis of variance (PERMANOVA). The envfit function was used to fit peptide variables to the ordination plot. Ellipses indicate 1 standard error from centroid. Arrow lengths are scaled at 0.5. Only peptides that showed significant correlation (*P* < 0.05) with the unconstrained ordination axes were plotted

As expected, age had an effect on the piglet fecal microbiomes at day 1, day 21 and day 35 (PERMANOVA *F* = 38.94, R2 = 0.37, *P* < 0.001) and samples (n = 136) showed distinct clusters by age (Fig. 4, stress = 0.093, k = 2). Peptides related to the sample plot distributions were fitted to the ordination using regression. IGF-1 (R2 = 0.61, *P* < 0.001), GIP (R2 = 0.20, *P* < 0.001), GLP-1 (R2 = 0.51, *P* < 0.001), and GLP-2 (R2 = 0.21, *P* < 0.001) were significantly associated with the piglet fecal community NMDS, while serotonin showed no significant association (R2 = 0.03, *P* = 0.15). Higher plasma concentrations of GLP-1 and GLP-2 were associated with day 1 fecal communities, while day 35 fecal samples were more closely associated with higher plasma levels of IGF-1. Plasma GIP levels had the strongest relationship primarily with fecal communities ordinated with the day 21 cluster.

### ASVs associated with plasma peptides and weight gain

**Fig. 5:**
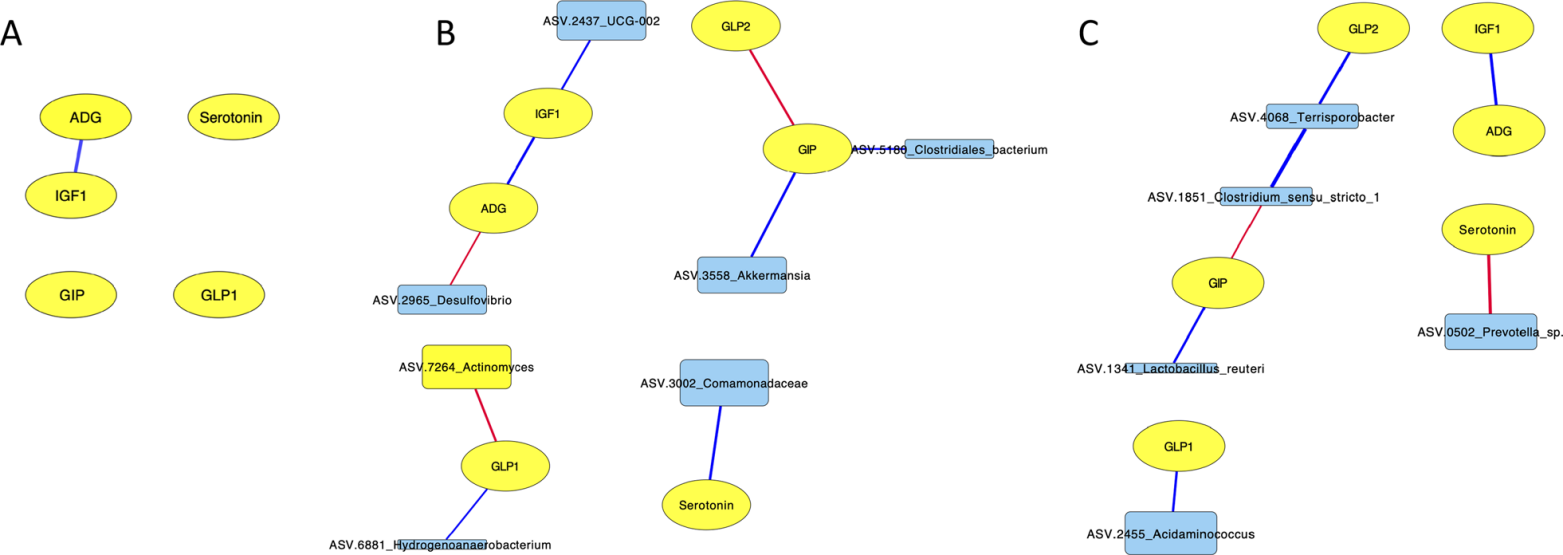
**A**–**C** Network plots of microbiota associations with peptides and average daily gain (ADG). Legend: Network plots showing direct associations among ASVs, peptides and ADG at time points (**A**) day 1, (**B**) day 21 and (**C**) day 35 using the FlashWeave program. Only significant associations between ASVs and metadata variables are shown (*P* < 0.01, FDR corrected). Edge color indicates association: negative (red), positive (blue); edge thickness indicates association weight. ASV node size is proportional to the mean centered log ratio abundance

Direct associations between ASVs in the piglet fecal microbiome and peptide concentrations or ADG were determined using a network-based approach for each time point at day 1, day 21 and day 35 (Fig. 5). Weights for each association are found in Additional file 2. Only a limited number of relationships were discovered, therefore the significant relationships between gut peptides and the microbiome were extracted from the overall networks at each age for ease of visualization. At day 1, no significant associations between fecal taxa and gut peptide concentrations or weight gain were determined; however, there was a positive association between IGF-1 and weight gain at day 1 of age. Day 21 microbiomes showed the most direct associations between taxa and peptides or weight gain (2 negative, 5 positive) including a positive association between an unclassified species from genus UCG-002 (family *Oscillospiraceae*) and IGF-1, positive associations between an unclassified *Akkermansia* species and GIP as well as between *Clostridiales* and GIP. Negative associations were identified between an unclassified *Desulfovibrio* species and weight gain, while GLP-1 was negatively associated with *Actinomyces* at day 21 of age.

At day 35, 3 positive associations and 2 negative associations between taxa and peptides were found. GIP was positively associated with *L. reuteri* and negatively with *Clostridium* sensu stricto 1. GLP-1 was positively associated with *Acidaminococcus* at day 35 of age. For all time points, peptide IGF-1 and weight gain were positively associated with each other.

## Discussion

Growth in the pig is characterized by a moderate preweaning growth rate with competition for nutrients amongst littermates between birth and weaning and dependent on the lactational capacity of the sow. This is often followed by a post weaning lag in growth rate, which was seen in this study between day 21 and day 24 of age when the average daily gain dropped by almost half during a period of major changes in the gut microbiome and gut permeability associated with a transition to a corn/soy based diet from the milk based diet. Successful transition to the weaning diet between day 24 and day 28 resulted in a rapid increase in average daily weight gain to compensate for the reduced energy intake and consequent reduced growth rate immediately following weaning (day 21 to day 24).

As mentioned, *C. scindens* was unique in the preweaning microbiome with association with preweaning growth. The high correlation of *C. scindens* with preweaning weight gain suggests a beneficial role for *C*. *scindens* in net nutrient uptake from the gut. *C*. *scindens* may interact with other components of the microbiome to promote net nutrient uptake or alter the gut ecology to augment gut health and consequent animal growth. Previous research has indicated that *C. scindens* can either directly inhibit the growth of *C. difficile* or indirectly inhibit *C. difficile* growth through secondary transformation of bile acids by hydroxylation [39, 40], and thus, promote overall gut health. In addition, these 7-hyroxylated bile acids can function as signaling molecules [41], for example as agonists for the Takeda G-protein receptor 5 (TGR5) and may indirectly contribute to the regulation of energy metabolism and inflammation [42]. *C. scindens* has also been demonstrated to induce GLP-1 expression; GLP-1 has an insulinotropic effect which suggests a promotion of net energy accretion in the pig [22]. Therefore, these data suggest *C*. *scindens* may serve as a potential marker for a gut microbial community beneficial to growth of the preweaning pig. The correlation between *C*. *scindens* and weight gain was gone after day 21 as the microbiome adapted to the dietary and physiological changes that occur with weaning. This study utilized the Illumina MiSeq platform, targeting the V4 region of the bacterial 16S rRNA gene. This region has been accepted as the most accurate target for bacterial taxonomy [25]. A caveat of this study is that the microbiome field continues to evolve through advances in sequencing technology, such as full-length 16S rRNA gene sequencing and metagenomics. While full-length 16S rRNA gene sequencing can allow for better taxonomic resolution, and thus, more accurate compositional data, the cost remains restrictive [43, 44]. Metagenomics, which investigates the total nucleotide sequences in a sample, often misses numerically inferior species and cannot investigate important members of the rare biosphere without additional targeted techniques [45, 46]. As costs continue to decrease for sequencing techniques, and updates are made to bacterial genome and pathway databases, microbiome data will become even more informative.

The changes in weight gain accompanied very large changes in microbiome dynamics following weaning. The reduction in weight gain between day 21 and day 24 following weaning was accompanied by both negative and positive correlations between weight gain and specific bacterial taxa. The high correlation between *Prevotellaceae* and weight gain was relatively unexpected due to the large reduction in growth rate between these ages and a presumed reduction in feed intake. The changes in the bacterial community as a result of the transition from a diet comprised of milk oligosaccharides used by *Bacteroidaceae,* prior to weaning, to the complex polysaccharides utilized by *Prevotellaceae* post-weaning has been previously reported [47–49] and may account for the large negative and positive correlations with weight gain observed for these two members of the bacterial community, respectively, between day 21 and day 24 of age. The positive association with *Prevotellaceae* may also be the consequence of the unique mucin glycoprotein degrading sulfoglycosidase that permits it to grow during the early weaning transition [50]. Following the transition to the complex carbohydrate based post-weaning diet, no correlations between *Prevotellaceae* and weight gain were detected at day 28 when weight gain had improved, suggesting that *Prevotellaceae* may be an early adapter to changes in diet in these swine. However, other members of the bacterial community also contribute in the present study during the transition to a more complex grain based diet as demonstrated by the positive correlations of *Veillonellaceae, Butyricicoccaceae, Spirochaetaceae, Muribaculaceae* and *Oscillospiraceae* with weight gain between day 21 and day 24*. Veillonellaceae, Butyricicoccaceae, Spirochaetaceae* and *Oscillospiraceae* are all short chain fatty acid producing bacterial families [51, 52] and in the present study may be important in the early adaption to metabolizing complex polysaccharides from grain starches. *Muribaculaceae* has been a largely uncharacterized clade, but whose abundance has recently been associated with an increase in fecal proprionate concentrations in mice [53] and swine [54], and genomes assembled from metagenomes indicate *Muribaculaceae* may express the necessary metabolic pathways to ferment polysaccharides into propionate, acetate and succinate [55]. *Rikenellaceae* RC9 has been associated with negative impacts on health and growth in several studies in mice [56–58], while the present study suggests a positive association with growth during the weaning adaptation.

The time from day 24 to day 28 demonstrated a rapid increase in weight gain, indicating the pigs’ successful adaption to the postweaning diet. This timespan was characterized by a smaller number of bacterial families correlating with weight gain; these correlations with weight gain trended weaker with only *Lactobacillaceae* and *Ruminococceae* correlations improved with ADG between day 24 and day 28 from day 21 to day 24. A different milieu of bacteria genera and species accounted for positive and negative correlations with body weight gain between day 24 and day 28 than those during the period between day 21 and day 24, with *Blautia* and *L. reuteri* demonstrating the highest positive correlations with body weight gain. *Blautia* has previously been correlated with increases in postweaning feed intake in swine [59], which can contribute to an increase in ADG; while specific *Lactobacillus reuteri* strains have been demonstrated to have probiotic properties in vitro [60, 61] and by oral administration to alter the composition of the microbial community within the gut of the newly weaned pig [62] as well as promote an increase in ADG in newly weaned pigs by enhancing intestinal barrier function [63]. However, the *L. reuteri* strains in the present study have not been identified. C*hristensenellaceae* R-7 were the only genus to have a negative correlation with ADG between day 24 and day 28. Previous research [64, 65] has indicated that unclassified members of C*hristensenellaceae* are associated with higher feed efficiency in swine post-weaning and function to maintain gastrointestinal tract structure and function, which suggests that further study is necessary to elucidate the specific role of C*hristensenellaceae* R-7 relative to other genera of C*hristensenellaceae* in gut health and animal growth*.* The large reduction in the number of negative correlations between taxa from day 21 to day 24 relative to day 24 to day 28 suggests that the pig successfully transitions to the Phase 1 diet by no later than day 28 of age as only one taxa is negatively associated with growth rate in that analysis group.

Weight gain continued to increase from day 28 to day 35 as the pigs grew more rapidly than earlier ages, suggesting the swine had successfully adapted to the dietary and environmental changes associated with weaning. Pigs were transitioned from nursery diet 1 to nursery diet 2 at day 28. This change in nursery diets follows standard practice in the swine industry to reduce feed costs by eliminating the highly digestible sprayed dried plasma proteins and reducing the amount of calf milk replacer found in the weanling diet, substituting soybean meal as a protein source and increasing the amount of corn in the diet as an energy source. Correlations between ADG and the microbial community experienced further changes during this timeframe, coinciding with the transition from nursery diet 1 to nursery diet 2. Previous research has suggested that unidentified *Prevotella* and *Faecalibacterium* can respond to dietary protein [66] and thus may account for their positive association with weight gain. Further research is needed to identify if soy protein can affect microbiome composition with phased nursery diet feeding.

The pathway analysis provided a few insights into the relationship of the microbiome to plasma peptides that can originate from the gastrointestinal tract and associated organs of the digestive system. The newborn gut ecology is undergoing significant changes with the introduction of dietary milk and exposure to the extrauterine environment, so relationships between the gut peptides and the microbiome at day 1 of age were not expected. However, the analysis demonstrated that GLP-1 and GLP-2 were both associated with the day 1 piglet fecal community microbiome. Both peptides were at their highest plasma concentrations at day 1 when comparing the three sampling ages. GLP-1 and GLP-2 both respond to nutrient ingestion and absorption [67, 68] and the response to the initial nutrient presentation during colostrum feeding may induce a maximal secretory response. Therefore, the association of plasma GLP-1 and GLP-2 with the day 1 fecal microbiome may be the consequence of the initial interactions of the adapting and developing microbiome with the digesta from colostrum and the subsequent absorption of simple sugars and lipids through the intestine or these nutrients may be bypassing the microbiome for direct absorption. IGF-1 was not associated with the microbiome at day 1 but was correlated with body weight. Analysis of plasma IGF-1 was included in this study due to its known associations with changes in body weight with age [69]. Previous research has shown that IGF-1 in newborn healthy humans is correlated to body weight [70], however the only study in swine to examine this relationship compared normal and growth retarded newborn pigs, finding a negative association [71], and no analysis was performed within the normal sized piglet group.

Plasma GIP concentration was associated with the day 21 fecal microbiome community. The microbiome at day 21 is much more complex that in the newborn pig [1, 4, 5, 11] and is fully capable of metabolic functions that produce numerous microbial metabolites that can communicate with the enteroendocrine system [15, 16]. Butyrate is one microbial metabolite that has been demonstrated to stimulate GIP release [19]. GIP functions to stimulate insulin secretion by the pancreas and to promote lipid accretion in adipose tissue. The neonatal pig is born with limited fat stores and rapidly accumulates adipose tissue lipids through weaning [72]. Thus, GIP may function in the preweaning pig to stimulate lipogenesis by adipose tissue in response to interactions with the microbiome.

Association of plasma IGF-1 with the microbiome has been previously reported [73]. Yan et al. [74] colonized the gut of germ-free mice and found an increase in serum IGF-1 and bone growth, while providing short chain fatty acids to antibiotic treated mice restored growth rates and increased serum IGF-1. If probiotics can be developed to specifically target microbial signaling for IGF-1 release, the potential for improving efficiency of production is significant as promotion of lean, fast growth is always a priority for the swine industry. Demonstration of an association of specific taxa with specific gut peptides in the present study suggests the potential for developing such probiotics that may result in a specific enteroendocrine response. However, further studies are necessary to elucidate these specific relationships.

## Conclusions

Demonstration of an association of certain taxa with individual gut peptides at specific ages suggests the potential for the microbiome to elicit changes in the gut enteroendocrine system during early postnatal development in the pig.

## Supplementary Information

**Additional file 1.** Nursery diet composition.

**Additional file 2.** Analyses of ASVs, time points, and animal weight by Spearman rank correlation.

## Data Availability

All sequences are publicly available in the NCBI’s Sequence Read Archive (SRA) under BioProjects PRJNA613280 (https://www.ncbi.nlm.nih.gov/bioproject/?term=PRJNA613280) and PRJNA673841 (https://www.ncbi.nlm.nih.gov/bioproject/?term=PRJNA673841).

## Abbreviations

ADG: Average daily gain
ASV: Amplicon sequence variant
CLR: Centered-log-ratio
CSS: Cumulative sum scaling
FDR: False discovery rate
g: Grams
GIP: Glucose-dependent insulinotropic polypeptide
GLP-1: Glucagon-like peptide 1
GLP-2: Glucagon-like peptide 2
IGF-1: Insulin-like growth factor 1
kg: Kilogram
NMDS: Non-metric multidimensional scaling
PERMANOVA: Permutational analysis of variance
TGR5: Takeda G-protein receptor 5
