# Supplementary material for: Enteroendocrine peptides, growth, and the microbiome during the porcine weaning transition
Source: Anim Microbiome. 2022 Nov 18;4:56. doi: 10.1186/s42523-022-00206-8 (PMC9673406; doi:10.1186/s42523-022-00206-8)
Supplement: Supplementary file 1 — Additional file 1. Nursery diet composition. [file 42523_2022_206_MOESM1_ESM.docx]

Additional File 1: Nursery Pig Diets

|  | Nursery 1 | | | | Nursery 2 | | |  | |
| --- | --- | --- | --- | --- | --- | --- | --- | --- | --- |
|  |  | |  | | |  | | |  |
| Age, Weeks | | 3 | | 4-6 | | |  |  |  |
|  | |  | |  | | |  |  |  |
| Ingredient | | % | | % | | |  |  |  |
| Corn | | 42.81 | | 51.40 | | |  |  |  |
| Milk Replacer | | 27.5 | | 10 | | |  |  |  |
| SBM | | 17.5 | | 32.02 | | |  |  |  |
| SDPP | | 5 | | 0 | | |  |  |  |
| Fat | | 0 | | 0.77 | | |  |  |  |
| Fish Meal | | 5 | | 3 | | |  |  |  |
| Dical P | | 0.38 | | 0.79 | | |  |  |  |
| Limestone | | 0.64 | | 0.81 | | |  |  |  |
| Salt | | 0.2 | | 0.25 | | |  |  |  |
| Zinc Oxide | | 0.375 | | 0.25 | | |  |  |  |
| Vitamins | | 0.25 | | 0.25 | | |  |  |  |
| Minerals | | 0.15 | | 0.15 | | |  |  |  |
| Methionine | | 0.09 | | 0.09 | | |  |  |  |
| Lysine | | 0.1 | | 0.2 | | |  |  |  |
| Threonine | | 0 | | 0.02 | | |  |  |  |

Calculated Analyis

Energy, ME kcal/kg 3520 3400

Crude Protein, % 24.74 24.08

Total Lysine % 1.50 1.35

Calcium, % 0.65 0.80

Phosphorus, total % 0.48 0.65
